# Targeting XBP1-mediated β-catenin expression associated with bladder cancer with newly synthetic Oridonin analogues

**DOI:** 10.18632/oncotarget.10863

**Published:** 2016-07-27

**Authors:** Wei Chen, Jiancheng Zhou, Kaijie Wu, Jun Huang, Ye Ding, Eun-Jin Yun, Bin Wang, Chunyong Ding, Elizabeth Hernandez, John Santoyo, Haiying Chen, Ho Lin, Arthur Sagalowsky, Dalin He, Jia Zhou, Jer-Tsong Hsieh

**Affiliations:** ^1^ Department of Urology, The First Affiliated Hospital, Medical School of Xi'an Jiaotong University, Xi'an 710049, China; ^2^ Department of Urology, University of Texas Southwestern Medical Center, Dallas, TX 75390, USA; ^3^ Department of Urology, Shaanxi Provincial People's Hospital, Xi'an, Shaanxi 710068, P.R. China; ^4^ Department of Pharmacology and Toxicology, University of Texas Medical Branch, Galveston, TX 77555, USA; ^5^ Department of Life Sciences, National Chung Hsing University, Taichung 40705, Taiwan; ^6^ Graduate Institute of Cancer Biology, China Medical University Hospital, Taichung 40447, Taiwan

**Keywords:** Oridonin, Wnt pathway, β-catenin, XBP1, transitional cell carcinoma

## Abstract

Conventional chemotherapy is commonly used for advanced stages of transitional cell carcinoma (TCC) with modest success and high morbidity; however, TCC eventually develops resistance. Muscle invasive bladder cancer (MIBC) is recognized as a lethal disease due to its poor response to traditional chemotherapy. Numerous studies have implicated β-catenin, a critical effector in Wnt–mediated pathway associated with epithelial-mesenchymal transition and cancer stem cell, is involved in TCC progression, and furthermore closely associated with chemo-resistance. In this study, we discovered a novel natural product analogue CYD 6-17 that has a potent inhibitory effect on TCC cells exhibiting drug resistance to various chemotherapeutics, with an IC_50_ at nM range. Delivery of CYD 6-17 significantly inhibited the tumor growth using xenograft model but without detectable side effects. Mechanistically, it targeted β-catenin gene transcription by decreasing the binding of XBP1 to the promoter region, which appeared to be a new regulatory mechanism for β-catenin gene expression. Clinically, XBP1 expression correlated with the poor overall survival of patients. Overall, this study unveils unique mechanism of β-catenin gene regulation in advanced TCC and also offers a potential rational therapeutic regimen to MIBC.

## INTRODUCTION

Transition cell carcinoma (TCC) is one of the most deadly cancers worldwide, particularly, advanced stages of TCC [1]. Despite intensive local therapy, patients with locally advanced bladder cancer are still at high risk for metastases. Conventional cytotoxic chemotherapy such as platinum based combination chemotherapy is commonly used as a main regimen for advanced stages of TCC, which currently provides the potential for cure only in selected patients and is underpowered [2]. Despite tremendous efforts, only small improvement on the overall survival of patients has been made in the treatment of advanced TCC over the past decade, and the long-term survival remains low [3]. In addition, frequent emerging of drug resistance due to tumor heterogeneity and serious side effects from current treatment regimens make treatment outcomes unsatisfactory. It is imperative to identify specific pathway(s) or molecular target(s) associated with advanced TCC that can achieve better therapeutic efficacy, while decreasing the morbidity and mortality related to TCC.

We recently developed an isogeneic TCC tumor model representing tumor progression from primary tumor to metastatic sites. The sublines derived from this model exhibit drug resistance to current chemotherapeutics commonly used in advanced TCC [4], which may possess stemness [5]. TCC is well recognized as a heterogeneous tumor. In advanced TCC, a subpopulation has been identified as cancer initiating cell or cancer stem cell (CSC) that also exhibits chemo-resistance leading to cancer mortality [6]. Global gene expression profiling of the CSCs revealed an activated gene-signature similar to that of aggressive TCC, supporting the concept that this subpopulation contributes to the progression [7, 8]. Several pathways, including Wnt, PI3K/Akt, and NF-κB have been implicated in CSCs related tumor chemo-resistance [9]. Very likely, a better treatment outcome could be achieved by targeting theses pathways.

It is known that natural products tend to target multiple mechanisms leading to tumor inhibition. Oridonin is a naturally occurring 7,20-epoxy-*ent*-kaurane diterpenoid isolated from *Isodon rubescens* traditionally used in China and Japan for the treatment of various human diseases, including cancer; it can target gene transcription [10]. In our study, we screened several analogues of Oridonin (i.e. CYD compounds) onto several drug-resistant TCC cell lines to identify potent candidates that can overcome drug resistance. CYD 6-17 appeared to be very potent to all the cell lines tested at a similar dosage range (i.e., nM) to induce apoptosis. By delineating its mechanism of action, we demonstrated that CYD 6-17 was able to suppress Wnt pathway by targeting β-catenin expression that has been shown to be associated with chemo-resistance of TCC and other cancers [11–18]. This study provides a strong rationale of applying CYD 6-17 as a new therapeutic agent onto advanced TCC patients.

## RESULTS

### Screening of CYD compounds for the growth inhibition of TCC

**Figure 1:**
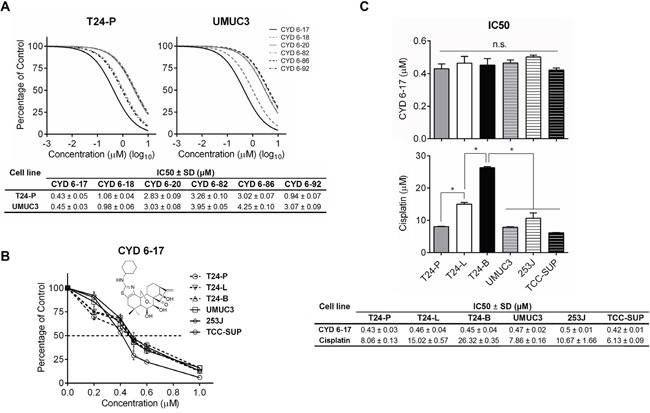
The effect of CYD compounds on TCC cell lines **A.** T24-P and UMUC3 cells were treated with different concentrations of CYD compounds and the relative cell number was determined 24 h after treatment using MTT assay (Upper panel) and IC_50_ was calculated and listed (Lower panel). **B.** The dose effect of CYD 6-17 (insert: chemical structure depicted) on TCC cell lines was determined by MTT assay. **C.** IC_50_ of CYD 6-17 and Cisplatin for each TCC cell line. Data represented mean ± SD (n = 3). *, *p* < 0.05.

Chemotherapeutic agents commonly used in TCC patients are targeting DNA proliferation and cell cycle [19, 20]. Eventually, TCC cells develop resistance to these agents. We decided to examine new agents with different mechanisms of action. Initially, we screened a variety of Oridonin analogues synthesized in-house using drug-resistant TCC cell lines (i.e., T24-P and UMUC3) (Figure 1A) and identified CYD 6-17 as the most potent compound with IC_50_values of 0.43±0.05 μM and 0.45±0.03 μM, respectively. Furthermore, we examined the effect of CYD 6-17 on a panel of TCC cell lines, including T24-P, T24-L, T24-B, UMUC3, 253J, and TCC-SUP. As shown in Figures 1B and 1C, CYD 6-17 was able to inhibit the cell growth of these TCC cell lines with similar IC_50_ (~ 0.5 μM) that appeared to be more sensitive than Cisplatin, and other chemotherapeutic drugs such as doxorubicin, Gemcitabine, and Mitomycin C (Supplemental Table S2). Thus, by further delineating its mechanism of action, we believe that CYD 6-17 has the potential to develop a new therapeutic agent for TCC patients.

### The biologic effect of CYD 6-17 on TCC cells

**Figure 2:**
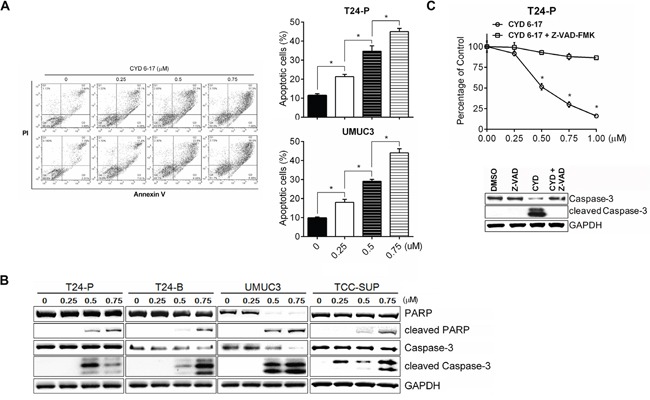
Induction of apoptosis by CYD 6-17 in TCC cell lines **A.** The cell death of T24-P and UMUC3 cells were determined 18 h after treatment using PI/Annexin V assay (Left panel) and the degree of cell apoptosis as depicted (Right panel). **B.** Western blot analyses of the expression of apoptotic markers (cleaved PAPR, cleaved Caspase-3) in TCC cell lines after treatment. **C.** Effect of Caspase inhibitor on apoptosis induced by CYD 6-17. T24-P cells were pretreated with pan-Caspase inhibitor Z-VAD-FMK (50 μM) for 2 h, followed by DMSO or CYD 6-17 (0.5 μM) treatment for 24 h. Data represented mean ± SD (n = 3). *, *p*< 0.05.

To understand the biological effect of CYD 6-17 on TCC cells, we determined whether CYD 6-17 was able to elicit apoptosis in T24-P and UMCU3 cells. As shown in Figure 2A, the incremental concentration of CYD 6-17 was able to induce cell apoptosis in a dose-dependent manner. The induction of apoptosis by CYD 6-17 treatment was further confirmed in several TCC cell lines based on the apoptotic markers cleaved Caspase-3 and poly ADP-ribose polymerase (PARP) (Figure 2B). However, when pretreated cells with pan-Caspase inhibitor Z-VAD-FMK, the apoptotic effect of CYD 6-17 significantly diminished (Figure 2C), indicating that the cytotoxic effect of CYD 6-17 on TCC cells is manly medicated through Caspase-dependent apoptosis pathway.

### Key mechanism of CYD 6-17 in TCC cells in inhibiting β-catenin gene expression

**Figure 3:**
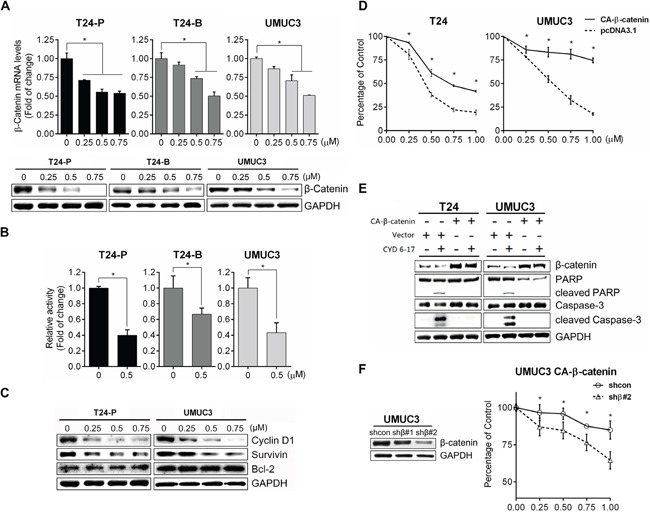
Effect of CYD 6-17 on β-catenin-mediated Wnt pathway **A.** Effect of CYD 6-17 on the expression of β-catenin mRNA (Upper panel) and protein (Lower panel) in T24-P, T24-B and UMUC3 cellsafter treatment. **B.** Effect of CYD 6-17 on β-catenin gene transcriptionactivityinT24-P, T24-B and UMUC3 cells transfected with TOP luciferase reporter gene. After normalizing with *Renilla* luciferase activity, the relative reporter gene activity in each cell line was calculated based on DMSO (=1). Data represented as mean ± SD (n=3). **C.** Western blot analyses of Bcl-2 and β-catenin downstream targets (CyclinD1 and Survivin) in TCC cell lines after treatment. **D.** Viability of CA-β-catenin or vector transfected T24 and UMUC3 cells after CYD 6-17 treatment was determined using MTT assay. Error bars represent SD; (n = 3). **E.** Expression of β-catenin and apoptosis markers (cleaved Caspase-3 and PAPR) from T24 and UMUC3 cells transfected with CA-β-catenin or vector then treated with CYD 6-17 (0 and 0.5 μM) for 24 h. **F.** Effect of β-catenin shRNAon TCC cells expressing CA-β-catenin treated with CYD 6-17. Left panel: Effect of shRNAs on the protein expression of β-catenin. Right panel: Effect of β-catenin shRNA on the viability of UMUC3 expressing CA-β-catenin treated with CYD 6-17.

To gain insight of molecular mechanism of CYD 6-17 in TCC cells, microarray analysis was carried out to profile its transcriptional regulation of mRNA expression. We identified 102 genes with differential expression (*p*<0.005) (Supplemental Figure S1A). Among them, β-catenin and several key downstream targets of Wnt-mediated pathway such as ABCG1, and ABCG5, were significantly suppressed. Since β-catenin plays an essential role in canonical Wnt-mediated pathway, we determined β-catenin mRNA levels after CYD 6-17 treatment and found the suppression of β-catenin mRNA and protein in a dose-dependent manner from several TCC cell lines (Figure 3A). This was confirmed by immunofluorescence in T24-P cells (Supplemental Figure S1B). Consistently, CYD 6-17 was able to suppress β-catenin-mediated gene transcription (Figure 3B). For example, decreased Cyclin D1 and Survivin protein levels, Wnt-regulated genes, were detected (Figure 3C), which confirmed the inhibitory effect of CYD 6-17 on Wnt-mediated pathway. However, Bcl-2 level, another key anti-apoptotic protein, did not alter in TCC cells treated with CYD 6-17, which was observed from leukemia cells treated with Oridonin [21]. This result indicated that CYD 6-17 may have substantially different mechanisms of action in TCC.

To determine whether the inhibition of β-catenin as the key mechanism of action of CYD 6-17 in TCC cell lines, we transfected constitutively active β-catenin (CA-β-catenin S37A) into T24 or UMUC3 (Supplemental Figure S2A) and the data demonstrated that the growth inhibitory effect of CYD 6-17 significantly diminished in both cell lines (Figure 3D). Consistently, the presence of CA-β-catenin in both cells was able to antagonize the apoptotic effect (cleaved Caspase-3 and PARP) elicited by CYD 6-17 (Figure 3E). By further knocking down β-catenin in UMUC3 cells expressing CA-β-catenin, cells regained their sensitivity to CYD 6-17 (Figure 3F).

### SUPPression of *in vivo* growth of TCC by CYD 6-17

**Figure 4:**
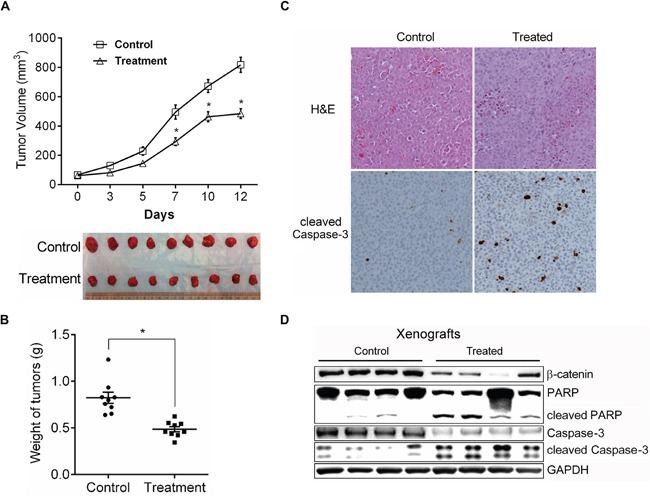
The *in vivo* effect of CYD 6-17 on tumor growth **A.** Effect of CYD 6-17 on the volume of TCC tumors. When the tumors became palpable, animals (n = 9 for each group) were randomized for CYD6-17 treatment. Tumor volume was measured every other day (Upper panel). All tumors were excised at Day 12 and photographed (Lower panel). **B.** Effect of CYD 6-17 on the weight of TCC tumors. *Significant difference between control and CYD 6-17 treatment group (*p*< 0.05). Error bars represent SD. **C.** Histologic analyses (200×) of TCC tumors after CYD6-17 treatment. Upper panel: H&E. Lower panel: IHC of cleaved Caspase-3. **D.** Effect of CYD 6-17 on apoptosis markers in tumors.

We therefore evaluated the anti-tumor efficacy of CYD 6-17 using subcutaneous xenograft model. The results showed that CYD 6-17 retarded the growth of tumors by 45% during the course of treatment (Figure 4A). The overall tumor weight from the treatment group was significantly lower than the control group (Figure 4B). Also, more apoptotic tumor cells were observed in the treatment group than the control group (Figure 4C). In addition, we were able to validate IHC result by determining the expression levels of apoptotic markers (Figure 4D).

### Molecular mechanism of CYD 6-17 in β-catenin gene regulation

**Figure 5:**
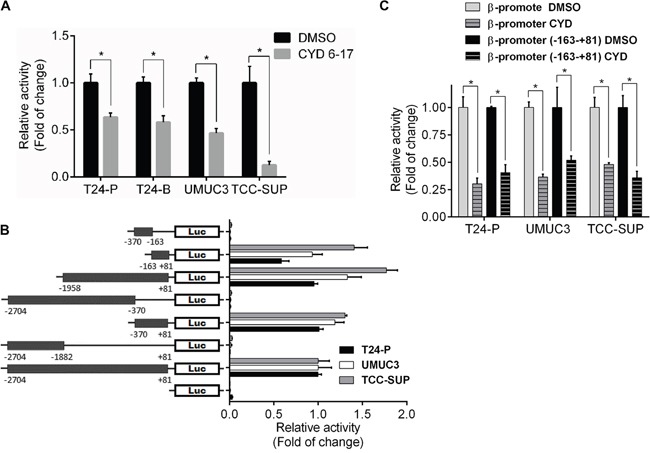
Effect of CYD 6-17 onβ-catenin gene promoter activity **A.** Effect of CYD 6-17 on β-catenin promoter activities in T24-P, T24-B, UMUC3 and TCC-SUP cells. After normalizing with *Renilla* luciferase activity, the relative reporter gene activity in each cell line was calculated based on DMSO (=1). **B.** Determination of core region of β-catenin gene promoter in TCC cell lines. Diagram represented each reporter gene construct containing different fragments of β-catenin gene promoter region. **C.** Effect of CYD 6-17 on “full-length” and core region of β-catenin gene promoter activities in T24-P, UMUC3 and TCC-SUP cells.

In presence of CYD 6-17, we noticed that endogenous β-catenin mRNA levels markedly decreased but exogenous CA-β-catenin mRNA expression driven by the CMV promoter had no significant change (Supplemental Figure S2B), implying the inhibitory effect of CYD 6-17 on β-catenin gene transcription. Indeed, the activities of β-catenin gene promoter were suppressed in several different TCC cell lines treated with CYD 6-17 (Figure 5A). We subsequently identified the core region (−163-+81) of β-catenin gene promoter responsible for its activities in T24, UMUC3 and TCC-SUP cells (Figure 5B). We further demonstrated that the main effect of CYD 6-17 on suppressing β-catenin gene promoter activities was on this core region as well (Figure 5C).

### The role of XBP1 in β-catenin gene transcription and its clinical correlation in TCC

**Figure 6:**
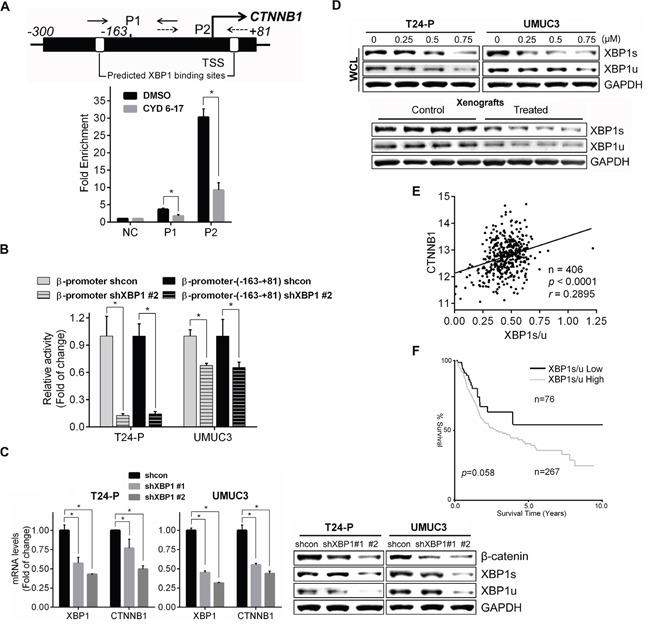
Regulation of β-catenin expression by XBP1 and its clinical relevance **A.** Effect of CYD 6-17 on the binding of XBP1 to β-catenin promoter region. Chromatin DNAs prepared from T24 cells treated with CYD 6-17 (0.5 μM) or DMSO for 10 h were subjected to ChIP. **B.** Effect of XBP1 on β-catenin gene promoter activity. T24-P and UMUC3 cells were transiently transfected with β-catenin reporter gene constructs promoters with shXBP1 construct then the luciferase activity was measured 36 h after transfection. After normalizing with *Renilla* luciferase activity, the relative reporter gene activity in each group was calculated based on shcon (=1). **C.** The expression of β-catenin mRNA (Left panel) or protein (Right panel) in T24-P and UMUC3 cells transfected with shXBP1 constructs. Data represented mean ± SD (n=3). *, *p* < 0.05.**D.** Effect of CYD 6-17 on the expression of XBP1 levels in whole cell lysate (WCL) in T24-P and UMUC3 12 h after treatment (Upper panel), or in tumor lysates (Lower panel). **E.** Correlation of XBP1s/u with expression of CTNNB1. The Pearson's correlation coefficient *r* value is shown. **F.** Kaplan-Meier curve analyses of XBP1s/u expression with overall survival of TCC patients. The log-rank test *p* value is shown.

To delineate what transcription factor(s) is responsible for the binding to this core region of β-catenin gene promoter in TCC cells, we used ALGGEN (http://alggen.lsi.upc.es/recerca/frame-recerca.html) to search transcription factors and XPB1 was found as a potential candidate that is further confirmed by the binding of XBP1 to this core region of β-catenin gene promoter and CYD 6-17 dramatically diminished its binding to this region (Figure 6A). Also, knocking down XBP1 significantly reduced the activities of both “full-length” and core region of β-catenin gene promoter (Figure 6B) as well as the expression of both β-catenin mRNA and protein (Figure 6C). Moreover, CYD 6-17 was able to decrease both XPB1 isoforms (i.e., XBP1u and XBP1s) in TCC samples from *in vitro and in vivo* (Figure 6D), and XBP1s decreased more substantially than XBP1u (Figures 6D and S2D). However, CYD 6-17 treatment did not alter total XBP1 mRNA level (Supplemental Figure S2C), implying CYD 6-17 may act on XBP1 post-transcriptionally. Collectively, these results indicated that XBP1 is the key transcriptional factor for regulating β-catenin gene expression and CYD 6-17 is able to target XBP1 protein, especially XBP1s from both *in vitro* and *in vivo* conditions.

It is known that XBP1s is a potent transcription factor and XBP1u acts as a negative regulator for XBP1s [22]. Thus, we decided to determine the clinical correlation of XBP1 in TCC based on XBP1s/u ratio. Indeed, a significant positive correlation between the expression of XBP1s/u and β-catenin (Figure 6E) as well as correlation between XBP1s/u and the overall survival of TCC patients (Figure 6F). Taken together, we conclude that a new mechanism of CYD 6-17 is to target XBP1-mediated β-catenin gene transcription in TCC cells and it appears to be a promising therapeutic agent for treatment of advanced TCC.

## DISCUSSION

The American Cancer Society estimated that ~16,000 persons will die with TCC in 2015 [1], indicating the continuous escalation of mortality. The mainstay of treatment of MIBC or metastatic TCC is conventional cytotoxic chemotherapy, in the form of MVAC (Methotrexate, Vinblastine, Adriamycin, and Cisplatin) or GC (Gemcitabine and Cisplatin). Although MVAC regimens result in overall response rates greater than 70%, the 5-year overall survival rates (13% for GC and 15.3% for MVAC) are poor [23]. Recent new regimen such as immune checkpoint inhibitors-Ipilimumab, a monoclonal antibody targeting CTLA-4, has shown an impressive early outcome of 18 % of patients survived beyond 2 years compared with a 5 % survival rate with the previous standard of care. However, a significant adverse events occur in >70 % of patients treated with Ipilimumab [24]. Obviously, there is clearly a need for 1) searching new potent agents that have different and unique mechanism of actions; 2) identifying key pathways or molecules underlying drug resistance.

In recent years, herbal medicine has drawn vast attention to become potential new cancer therapeutics due to its remarkable growth inhibition and low side effects [25]. Natural product analogues may act as promising agents capable of targeting multiple mechanisms that are often associated with tumor heterogeneity and progression, and likely, tumor cells have less chance to develop the resistance. Indeed, we noticed that CYD 6-17 appeared to be very potent (IC_50_ at nM range) to inhibit a variety of TCC cell lines possessing different genetic profiles and drug resistance to MVAC. The clinical development of Oridonin for cancer therapy has been hampered, to a large degree, by its relatively moderate potency, limited aqueous solubility and bioavailability [26]. Recently, we have developed novel synthetic methodologies to access structurally diversified diterpenoids, and designed a series of nitrogen-enriched Oridonin analogues [27]. CYD 6-17 is a thiazole-fused analogue at C-1 and C-2 of the A-ring, with enhanced anticancer potency and aqueous solubility, and even lower toxicity to normal cells in comparison with Oridonin [28]. The result from *in vivo* model also indicated no significant side effect associated with CYD 6-17 during the entire treatment course. We believe this compound has a clinical translational potential for the systemic application of advanced TCC patients.

To delineate molecular mechanism of CYD 6-17, our microarray analysis indicated that down-regulation of canonical Wnt pathway mediated by β-catenin appears on the top of list. In TCC, β-catenin has been demonstrated to play a critical role in tumorigenesis [29, 30]. And silencing of β-catenin by siRNA increased TCC cell apoptosis and cell cycle arrest [31], which is consistent with our findings. Although we observed decreased expression of β-catenin downstream target Survivin after CYD 6-17 treatment, which could partially explain the increased apoptosis and is different from previous report about the effect of Oridonin on leukemia, implying that CYD 6-17 has distinct mechanism of action from Oridonin in TCC, while the detailed association between β-catenin and apoptosis needs further investigation. Furthermore, CSC is implicated to be involved in chemo-resistance, metastasis, and tumor recurrence from many cancer types [4, 5]. Meanwhile, β-catenin has been implicated in the self-renewal of CSCs. Notably, in TCC specimens, nuclear β-catenin was expressed more restrictedly in the CD44^+^ tumor cell subpopulation [7], implying that the transcriptional activities of β-catenin play a key role in the maintenance of stemness. Recent data also demonstrated that CSC in TCC can contribute to the chemo-resistance [6]. In addition, the β-catenin downstream genes such as ABC family members are known to be responsible for multi-drug resistance, including Cisplatin [9]. Furthermore, this effect could be reversed by β-catenin knockdown by siRNA [12]. Overall, β-catenin plays a pivotal role in TCC tumorigenesis, progression and chemo-resistance. Targeting β-catenin is a promising strategy for advanced TCC therapy.

Although many different kinds of mutation including β-catenin and APC have been found in Wnt pathways from different cancer types [32], in TCC, epigenetic silencing of Wnt antagonists appear to be more common [33]. Also, gene mutations of β-catenin [34] and its negative regulator Adenomatous polyposis coli (APC)have been reported in TCC patients associated with activated Wnt pathway leading to poor overall patient survival [35]. To date, almost all the efforts to target Wnt/β-catenin pathway focus on protein interaction or enzyme activities [36], which may not be most effective way for TCC due to the existence of mutations in this pathway. Instead, CYD 6-17 represents a new class of agent that can effectively target β-catenin gene transcription via inhibiting XBP1.XBP1 is a key factor in endoplasmic reticulum (ER) stress and unfolded protein response (UPR) has been shown to play an important role in adaptive response of cancer cells to environmental stress such as chemotherapeutics [37]. However, the role of XBP1 in TCC is largely unknown. In general, XBP1 encodes two isoforms: XBP1s contains the nuclear localization signal and the transcriptional activation domain, whereas XBP1ucontains the nuclear exclusion signal. XBP1u has been shown to negatively regulate the transcription activity of XBP1s by forming a complex that is sequestered from the nucleus [22]. Thus, the value of XBP1s/u is an indicator of XBP1s activity; XBP1s/u as an independent prognostic marker, can be used to predict the overall survival of myeloma patients [38]. Here, for the first time in the literature, we were able to demonstrate the clinical correlation of elevated XBP1s/u with poor prognosis in TCC patients and its critical regulation of β-catenin gene expression. By further evaluating whether XBP1 can be an independent prognostic marker, we can expect to use this marker to stratify patients for CYD 6-17 as a targeted therapeutic agent. Most importantly, CYD 6-17 exhibits a unique mechanism of action to target β-catenin gene transcription by targeting XBP1 that has not been reported in literature. In conclusion, CYD 6-17 represents a potent therapeutic agent to overcome drug resistant TCC and it also provides a new understanding of functional role of XBP1 in TCC. Certainly, the detailed mechanism of XBP1 protein degradation requires further investigation.

## MATERIALS AND METHODS

### Cell culture, drugs and antibodies

T24, 253J, UMUC3 and TCC-SUP were purchased from the American Type Culture Collection (ATCC, Manassas, VA). Cells were maintained in T-medium (Invitrogen, Carlsbad, CA) supplemented with 5% fetal bovine serum and 1% penicillin/streptomycin in a humidified incubator at 37°C and 5% CO_2_. T24 and UMUC3 are high grade TCC, 253J invasive metastatic TCC, and TCC-SUP high-grade invasive TCC. Previously we have developed several sublines of T24 such as primary T24-P, lung-metastatic T24-L and bone-metastatic T24-B as described previously [4] and cultured in the same medium as T24, of which T24-L/B are more resistant to chemotherapeutic drugs than T24. Cell lines were authenticated using AmpFLSTR®Identifier® PCR Amplification kit (Applied Biosystems, Grand Island, NY) and MycoAlert® kit (LonzaWalkersville, Inc. Walkersville, MD) to confirm mycoplasma-free condition. All CYD compounds were prepared following our published synthetic procedures [27, 28, 39, 40], and dissolved in dimethyl sulfoxide (DMSO).

Primary antibodies used were: mouse monoclonal antibodies to Bcl-2, Survivin, Histone H1, α-tubulin and GAPDH (Santa Cruz Biotechnology, Santa Cruz, CA), rabbit antibody to Caspase-3 (BD Transduction Laboratories San Jose, CA), rabbit monoclonal antibodies to β-catenin, cleaved Caspase-3 and PARP (Cell Signaling Technology, Beverly, MA), rabbit polyclonal antibody to XBP1 (Santa Cruz Biotechnology, Santa Cruz, CA).

### RNA extraction, quantitative real-time RT-PCR (qRT-PCR), and microarray analysis

Total cellular RNA was extracted using Maxwell® 16 LEV simplyRNA Cells Kit (Promega,) and reversely transcribed with iScript™ Advanced cDNA Synthesis Kit for qRT-PCR (BioRad, Hercules, CA) according to the manufacturer's instructions. qRT-PCR was carried out in a 20 μL reaction volume in iCycler thermal cycler (BioRad) using SuperScript® III Platinum® SYBR® Green One-Step qRT-PCR Kit (Life Technologies). Gene-specific primers for 18S, β-catenin and XBP1 were listed in Supplemental Table S1. The relative mRNA expression was calculated after normalizing with 18S for each sample. Each treatment condition was performed in triplicates.

Cells were treated with DMSO or CYD 6-17 (0.5 μM) for 8 h. Biotin-labeled cRNA was prepared from 1 μg of total RNA, fragmented, and hybridized to Affymetrix human U133 plus 2.0 expression array. All gene expression microarray data were normalized with Z score transformation. Paired *t* statistical tests were used to identify the differentially expressed genes (*p*<0.005).

### Cell growth assay

Briefly, cells (3×10^3^) were seeded in 96-well plate. After 24 h, medium were aspirated and new medium containing different concentrations of drugs added; each treatment condition was performed in triplicate. After 24 h of incubation, cell growth was monitored using Cell Proliferation Kit I (MTT) (Roche, Indianapolis, IN) according to manufacturer's instruction. The absorbance was recorded at 550-570 nm by Epoch Microplate Spectrophotometer (BioTek, Winooski, VT). IC_50_ values were calculated with GraphPad Prism software.

### Plasmids and gene transfection

TOP-flash, FOP-flash, constitutively active β-catenin (S37A) (CA-β-catenin) and its control were obtained from Dr. Zijie Sun (Stanford University). Plasmids containing scrambled shRNA and shRNAs targeting β-catenin and XBP1were from Dr. Ho Lin (National Chung Hsing University, Taiwan). The oligosequences of the shRNAsare listed in Supplemental Table S1.

For gene transfection, 3×10^5^ cells were seeded in a P-60 dish (with 50–60% confluence) and transfected using jetPEI transfection reagent (PolyPlus-transfection, Illkirch, France) according to the manufacturer's instructions and cells were harvested after 48 h of transfection. For establishing stable transfectants, cells were selected with incremental G418 for 4 weeks.

### Construction of luciferase reporters driven by CTNNB1 (β-catenin gene)promoter or its fragments

For cloning the promoter of human β-catenin gene, a specific primer set (Supplemental Table S1) [41] was used to amplify genomic DNA isolated from SV-HUC cells using KAPA HiFiHotStartReadyMix PCR Kit (Kapa, Boston, MA) and cloned into pGEMT Vector (Promega, Madison, WI) then validated by DNA sequencing. The correct clone was subsequently inserted into KpnI-XhoI site of pGL4.14-luc2/Hygro (Promega). For constructing variable fragments of β-catenin gene promoter, different restriction enzymes were used to delete specific fragment then re-ligated and validated by sequencing.

### Dual-luciferase reporter assay

For determining gene promoter activities, 1 μg of reporter constructs and 2 ng of pRL-SV40 Renilla luciferase construct (as an internal control) were transfected into 3×10^4^ cells in 24-well plate. After 24 h of transfection, cell lysates were prepared by Passive Lysis Buffer, and the luciferase activity was measured using Dual-Luciferase^®^ Reporter Assay System (Promega) in Veritas™ Microplate Luminometer (Turner BioSystems, Sunnyvale, CA) as described previously [42]. Wnt-elicited gene transcription was measured by the ratio of TOP and FOP luciferase activity. β-catenin gene promoter activity was determined by the activity of each constructs subtracted from that from control plasmid (pGL4.14-luc2/Hygro). Relative luciferase activity represented as mean ± SD from each sample in triplicates after normalizing with the Renilla luciferase activity.

### Western blot

Total cellular protein lysates from culture cell or tumor were prepared with RIPA buffer (50 mM Tris [pH 8.0], 150 mM NaCl, 0.1% SDS, 1% NP40 and 0.5% sodium deoxycholate) containing proteinase inhibitors, 1% Cocktail and 1 mM PMSF (Sigma, St Louis, MO). For nuclear protein isolation, NE-PER Nuclear and Cytoplasmic Extraction Reagents (Thermo Scientific, Waltham, MA) was carried out for extraction. An equal amount of protein was separated by Bolt 4-12% Bis-Tris Plus Gels (Life Technologies) and transferred to nitrocellulose membranes. Following blocking with 5% skim milk in Tris-buffered saline with 0.1% Tween 20 (pH 7.6, TBST), the membranes were incubated with primary antibodies at 4°C overnight. After being washed with Phosphate Buffered Saline with Tween® 20, membranes were incubated with secondary antibodies coupled to horseradish peroxidase at room temperature for 1 h, then added SuperSignal West Dura Chemiluminescent Substrate (Thermo Scientific) and visualized with an Alpha chemiluminescent detection system (Pierce, Rockford, IL, USA). GADPH was used as an internal loading control for total cell lysate, Histone H1 for nucleus lysate and α-tubulin for cytosol lysate.

### Immunofluorescence staining

Cells were fixed in 4% paraformaldehyde for 30 min at room temperature and washed three times with PBS then permeabilized with 0.5% Triton-X100 for 20 min. The slides were blocked in 5%BSA containing 0.1% Triton-X100 for 1 h at room temperature, and then incubated with β-catenin antibody in 1:200 dilutions in PBS containing 1%BSA overnight at 4°C. After washing with PBS, cells were incubated with Alexa Fluor® 594 (Life Technologies) for 1 h at room temperature. Finally, cells were counterstained with ProLong® Gold antifade reagent (Thermo Scientific) before mounting.

### Flow cytometry

For detection of cell apoptosis status, cells were treated with DMSO or CYD 6-17 for 12 h, then determined by Dead Cell Apoptosis Kit (Life Technologies) and analyzed using flow cytometry (FACS Calibur, BD Biosciences).

### Chromatin-immunoprecipitation assay (ChIP assay)

ChIP assay was performed by using ChIP-IT Express Enzymatic kit (Active Motif, Carlsbad, CA) according to the manufacturer's instructions. Briefly, cells were cross-linked with 1% formaldehyde for 10 min, quenched with glycine followed by nuclear lysis. After isolating nuclear fractions, chromatin was enzymatically sheared into 150-600 bp. The sheared DNA was immunoprecipitated with ChIP-grade Antibodies overnight. After reversal of cross-linking, DNA fragments were purified on spin columns. The XBP1 binding sites in the CTNNB1 promoter were amplified by PCR from purified chromatin. Rabbit IgG (immunoglobulin G) was used as a negative control and a primer set for amplifying β-catenin promoter region without predicted XBP1 binding site was used as an internal control. The primers used in this experiment were listed in Supplemental Table S1.

### Mouse xenografts

All animal work was approved by the Institutional Animal Care and Use Committee. UMUC3 cells (1×10^6^) were subcutaneously injected into the flanks of six-week-old SCID mice. When tumor size reached 50-100 mm^3^, drug treatment started and tumor volume (cubic millimeters) was measured. For drug treatment, ALZET osmotic pumps (DURECT Corporation, Cupertino, CA) containing DMSO or CYD 6-17 were mounted subcutaneously beside xenograft tumors. The drug (30mg/kg/d) was delivered by pump continuously for 7 days, and then i.p. injection of 10 mg/kg/d was carried out for 3 additional days. Tumor volume was calculated by using the ellipsoid formula (π/6 × length × width × depth).

### Immunohistochemical (IHC) staining

Xenograft tumor tissues were fixed in 4% paraformaldehyde and performed immunohistochemistry on 5-μm-thick paraffin sections after heat-induced antigen retrieval. IHC was carried out by incubating the primary antibody for cleaved Caspase-3 (Cell Signaling Technology, 1:400) for 30 min at room temperature or mouse pre-immune serum as a control in a fully automated Ventana Autostainer model Discover XT ™ (Ventana Medical System, Tuscan, AZ) using Universal DAB Detcction Kit (Ventana Medical Systems). After counterstaining with haematoxylin, slides were mounted.

### Bioinformaticand statistical analyses

XBP1 and CTNNB1 expression in TCC samples were analyzed from The Cancer Genome Atlas (TCGA) database (http://cancergenome.nih.gov/). The value of XBP1s/u was calculated by dividing the expression values of spliced XBP1 (XBP1s) mRNA by un-spliced XBP1 (XBP1u) mRNA. Data was analyzed by GraphPad Prism software. The correlation between XBP1s/u and CTNNB1 was evaluated by Pearson correlation analysis. Data was visualized using a scatter plot and a solid line representing the linear regression line.

Survival status of the TCC patients from TCGA database was examined. X-tile, a tool for biomarker assessment and outcome-based cut-point optimization [43], was used to generate an optimal cut-off point to dichotomize XBP1s/u values as “High” and “Low”. Accordingly, patients were divided into two subgroups and the correlation of XBP1s/u with 10-years overall survival was evaluated. Kaplan–Meier survival analysis was performed and log-rank test was used to assess the statistical significance of survival difference between these two subgroups.

All error bars in graphical data represent mean ± SD. Student's two-tailed t-test was used for the determination of statistical relevance between groups, and p<0.05 was considered statistically significant. All statistical analyses were performed with GraphPad Prism software.

## SUPPLEMENTARY MATERIALS FIGURES AND TABLES


