# Supplementary material for: Targeting XBP1-mediated β-catenin expression associated with bladder cancer with newly synthetic Oridonin analogues
Source: Oncotarget. 2016 Jul 27;7(35):56842–54. doi: 10.18632/oncotarget.10863 (PMC5302956; doi:10.18632/oncotarget.10863)
Supplement: Supplementary file 1 [file oncotarget-07-56842-s001.pdf]

# Targeting XBP1-mediated β-catenin expression associated with bladder cancer with newly synthetic Oridonin analogues

## SUPPLEMENTARY TABLES AND FIGURES

**Supplementary Table S1: Primers for qRT-PCR, CTNNB1 promoter cloning and ChIP assay**

| **Primers for qRT-PCR** | | |
|---|---|---|
| **Gene** | **Forward** | **Reverse** |
| **18S** | GGAATTGACGGAAGGGCACCACC | GTGCAGCCCCGGACATCTAAGG |
| **CTNNB1** | ACTACTGTGGACCACAAGCAGAG | AGTCCCAAGGAGACCTTCCATC |
| **XBP1** | GGAGTTAAGACAGCGCTTGG | ACTGGGTCCAAGTTGTCCAG |

| **Primers for CTNNB1 promoter cloning** | |
|---|---|
| **Forward** | GCTGCGATGCGGTACCGAACGAACCTTCTTTACAATACTGGCATAT |
| **Reverse** | GCGACTCGATCTCGAGACGCTGCTGCCACAGACCGAGAGGCTTAAAAT |

| **Primers for ChIP assay** | | |
|---|---|---|
| **Set** | **Forward** | **Reverse** |
| **Control** | AAGCCTCTGTTTTAGCGAAGG | ACCCCGGGTTTGACATAAAT |
| **P1** | CCCGATGCAGACCACAG | CGCAGAAGCCGCTGTAT |
| **P2** | GCTCAGACGGCAGCAGAC | GACCGAGAGGCTTAAAATGG |

**Supplementary Table S2: $IC_{50}$ of TCC-SUP, T24, and UMUC3 for various chemotherapeutics (www.cancerrxgene.org)**

| Cell line | IC50 (µM) | | | | | |
|---|---|---|---|---|---|---|
| | Cisplatin | Doxorubicin | Vinblastine | Methotrexate | Mitomycin C | Gemcitabine |
| **TCC-SUP** | 5.5616 | 0.2455 | 0.0051 | 13.9548 | 0.2458 | 2.0763 |
| **T24** | 7.0357 | 0.0860 | 0.0079 | 12.7190 | 0.1018 | 0.0162 |
| **UMUC3** | 8.4988 | 1.2308 | 0.0236 | 8.5696 | 0.4817 | 1.1679 |

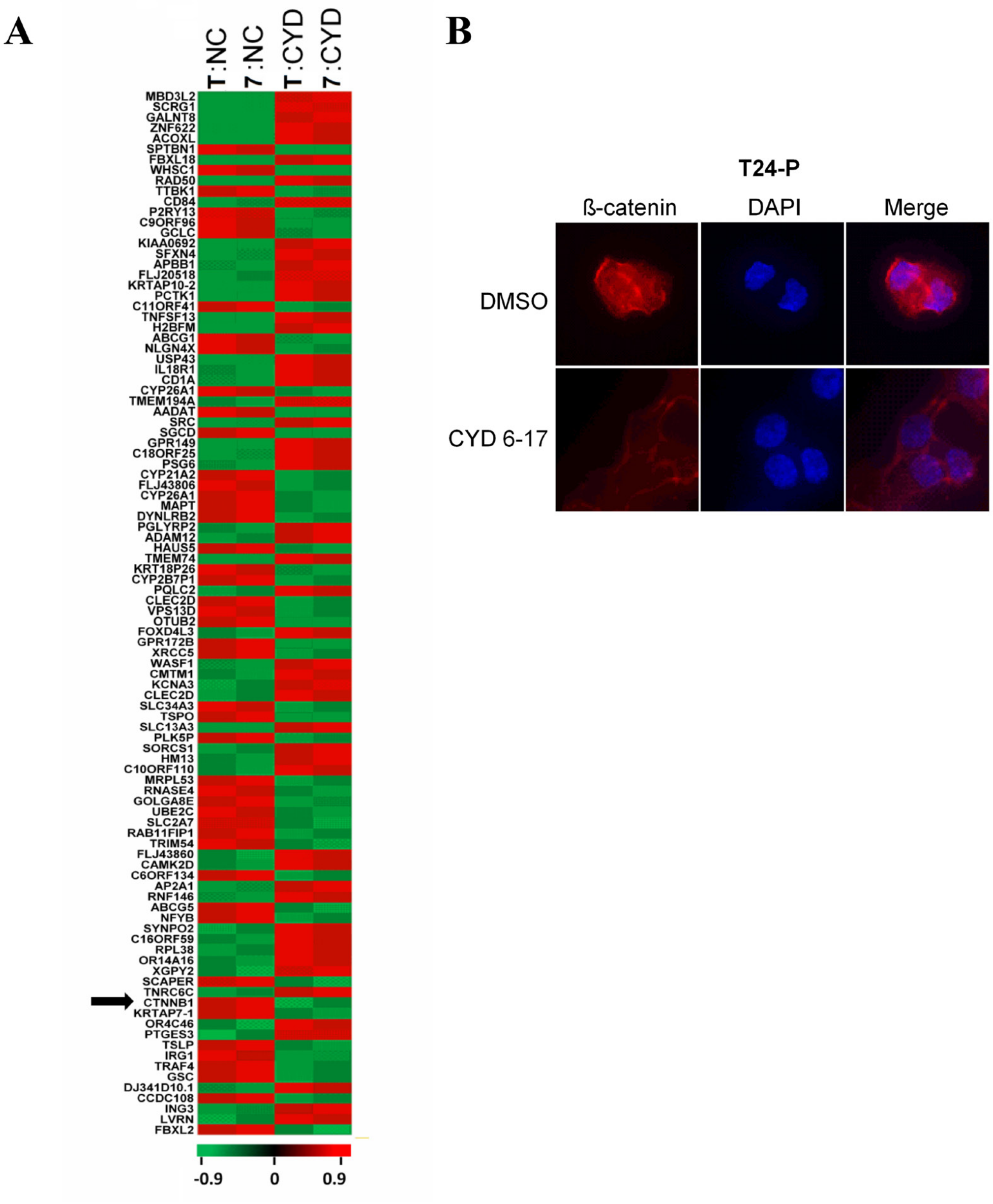


**Supplementary Figure S1: A.** Heat map of the gene expression profile regulated by CYD 6-17 treatment in T24-B and 786-O. Black arrow indicates CTNNB1. T: T24-B; 7: 786-O. NC: negative control; CYD: CYD 6-17 treated. Scale bar shown for Z score value. **B.** Immunofluorescence staining of β-catenin expression after CYD 6-17 treatment for 12 h in T24-P cells.

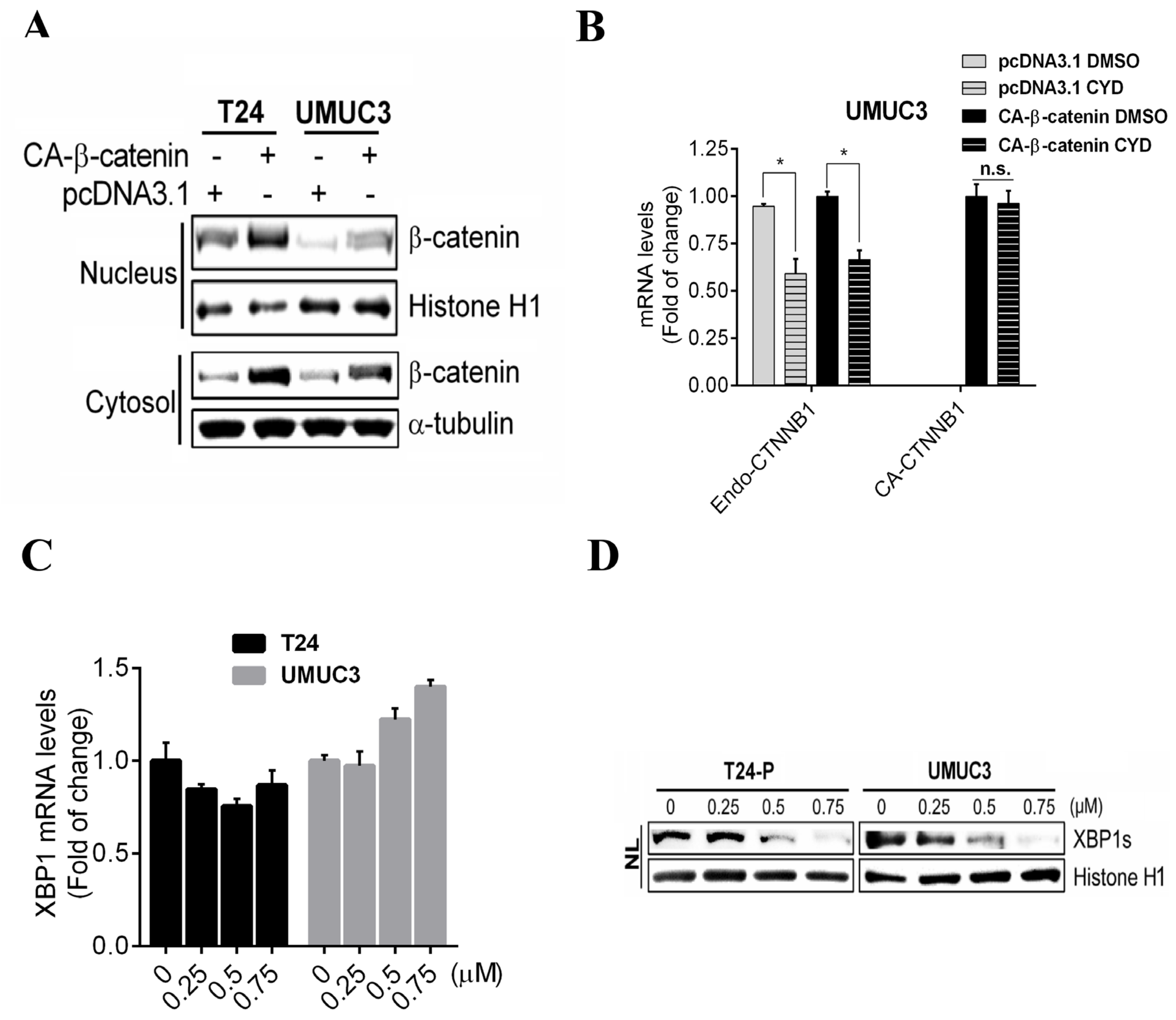


**Supplementary Figure S2: A.** Effect of CYD 6-17 on the mRNA expression from endogenous β-catenin orexogenous CA-β-catenin in UMUC3 cells. *, $p < 0.05$. **B.** XPB1 mRNA expression in TCC cells after CYD 6-17 treatment. **C.** Expression of β-catenin in nuclear and cytosol fractions in T24-P and UMUC3 cells transfected with CA-β-catenin or vector. **D.** Effect of CYD 6-17 on the expression of XBP1s in nuclear lysate (NL) in T24-P and UMUC3 12 h after treatment.
